# Functional Characterization of Two RNA Methyltransferase Genes *METTL3* and *METTL14* Uncovers the Roles of m^6^A in Mediating Adaptation of *Plutella xylostella* to Host Plants

**DOI:** 10.3390/ijms231710013

**Published:** 2022-09-02

**Authors:** Bei-Bei Wang, Ying-Fang Lai, Fei-Fei Li, Lu Jiao, Qing-Xuan Qiao, Shan-Yu Li, Xiu-Juan Xiang, Huang Liao, Min-Sheng You, Wei-Yi He

**Affiliations:** 1 State Key Laboratory for Ecological Pest Control of Fujian and Taiwan Crops, Institute of Applied Ecology, Fujian Agriculture and Forestry University, Fuzhou 350002, China; 2 International Joint Research Laboratory of Ecological Pest Control, Ministry of Education, Fujian Agriculture and Forestry University, Fuzhou 350002, China; 3 Ministerial and Provincial Joint Innovation Centre for Safety Production of Cross-Strait Crops, Fujian Agriculture and Forestry University, Fuzhou 350002, China

**Keywords:** *N*^6^-methyladenosine, RNA methyltransferase, host adaptation, *Plutella xylostella*

## Abstract

*N*^6^-methyladenosine (m^6^A) is one of the major epigenetic modifications in eukaryotes. Although increasing functions of m^6^A have been identified in insects, its role in *Plutella xylostella* L. for host plant adaptation remains unclear. In the current study, we show that the m^6^A content of *P. xylostella* was relatively low in different developmental stages and tissues, with no significant differences. Two RNA methyltransferase genes, *PxMETTL3* (methyltransferase-like 3) and *PxMETTL14* (methyltransferase-like 14), were identified and characterized. *PxMETTL3* could be transcribed into two transcripts, and *PxMETTL14* had only one transcript; both of these genes were highly expressed in egg and adult stages and reproductive tissues. The CRISPR/Cas9-mediated knockout of *PxMETTL3* (Δ*PxMETTL3-2*) or *PxMETTL14* (Δ*PxMETTL14-14*) confirmed their function in m^6^A installation into RNA. Furthermore, upon transfer from an artificial diet to the host plant, the mutant strains were affected in terms of larval and pupal weight or adult emergence rate, while the wildtype (WT) strain did not exhibit any difference. In addition, the fecundity and egg hatching rate of the WT strain decreased significantly, whereas only the Δ*PxMETTL14-14* mutant strain displayed significantly decreased fecundity. There seemed to be a tradeoff between the stress adaptation and reproduction in *P. xylostella* mediated by m^6^A modification. During host transfer, the expression of *PxMETTL14* was consistent with the change in m^6^A content, which implied that *PxMETTL14* could respond to host plant defense effectively, and may regulate m^6^A content. Kyoto Encyclopedia of Genes and Genomes (KEGG) pathway enrichment analysis of the differentially expressed transcripts with changes in m^6^A levels revealed that the potential functions of m^6^A-related genes may be involved in steroid biosynthesis for larval performance and metabolic pathways for adult reproduction. Overall, our work reveals an epigenetic regulation mechanism for the rapid adaptation of *P. xylostella* to variations in the host environment.

## 1. Introduction

Epigenetics usually refers to the alteration of DNA methylation, histone modifications, and chromatin accessibility that regulate gene expression in response to environmental challenges [1]. Numerous studies have identified and functionally characterized various RNA base modifications in protein-coding and noncoding RNAs, a field termed epitranscriptomics, which also result in changes in gene expression regulation [2]. *N*^6^-methyladenosine (m^6^A) is among the most abundant chemical modifications present in eukaryotes, including yeasts [3], plants [4,5], flies [6], and mammals [7,8,9], and is mainly located on mRNAs, tRNAs, rRNAs, lncRNAs, and miRNAs [10,11,12]. This kind of modification regulates important cytological processes, such as transcription, mRNA stability, RNA alternative splicing, protein translation efficiency, and X chromosome inactivation [13,14].

The m^6^A toolkit is composed of three classes of protein factors: “writers” (methyltransferases, covalently adding methyl groups at the N6 position of adenine), “erasers” (demethylases, making m^6^A a reversible reaction), and “readers” (m^6^A-binding proteins, recognizing the base undergoing m^6^A modification) [15]. m^6^A is installed into RNA by the “writer” complex consisting of two core components, methyltransferase-like 3 (METTL3) and methyltransferase-like 14 (METTL14), as well as some accessory factors [16,17]. m^6^A can be reduced to adenosine by the demethylation function of fat mass and obesity-associated protein (FTO) or AlkB homolog 5 (ALKBH5) [18,19]. m^6^A readers utilize different mechanisms to select and bind RNAs containing m^6^A sites, including YT521-B homology (YTH) domain-containing proteins, heterogeneous nuclear ribonucleoprotein (HNRNP) C/G (HNRNPC/G), HNRNPA2B1, insulin-like growth factor 2 mRNA binding protein 1-3 (IGF2BP1-3) and fragile X messenger ribonucleoprotein 1 (FMR1) [16].

m^6^A can regulate the alternative splicing of *Sxl* (sex-lethal) pre-mRNA and modulate flying behavior in *Drosophila melanogaster* Meigen [20,21], where the ‘‘reader’’ YT521-B is a major m^6^A effector [22]. The level of m^6^A in the insect vector *Laodelphax striatellus* Fallén was found to be negatively correlated with the replication of rice black-streaked dwarf virus (RBSDV) [23]. In *Bemisia tabaci* Gennadius, a mutation in the 5′ untranslated region (UTR) of a cytochrome P450 gene CYP4C64 introduces a potential m^6^A site, which confers resistance to the thiamethoxam [24]. The expression levels of *METTL3*, *METTL14*, and *fl(2)d* (female-lethal(2)d), and the content of m^6^A changed significantly across different stages of worker and queen bee larvae of *Apis mellifera* L., indicating that m^6^A functionally impacts caste differentiation and larval development [25]. Compared to the nondiapause-destined strain, the diapause-destinated strain of *Bombyx mori* L. has higher m^6^A content and m^6^A-related gene expression [26]. The modification of m^6^A in the RNA transcripts of *B. mori* was affected by nucleopolyhedrovirus infection [27]. Moreover, CRIPR/Cas9-based knockout of a single allele of the *WTAP* (Wilms’ tumor 1-associating protein) homolog *fl(2)d* in *Plutella xylostella* was reported to significantly decrease the fecundity and fertility of female adults [28].

The diamondback moth (*P. xylostella*) is an important pest that preferentially infests different cultivated vegetables and wild cruciferous plants [29,30]. Nonetheless, it has been observed that *P. xylostella* is able to establish a stable population on the noncruciferous vegetable pea (*Pisum sativum* L.) when the preferred host is absent [31]. Evidence has been provided that the DNA methylation of *P. xylostella* upon host transfer from *Raphanus sativus* L. to *Arabidopsis thaliana* L. undergoes reprogramming, which might influence adaptation-associated gene expression [32]. Therefore, it would also be interesting to explore the epitranscriptomic dynamics of this process, and the roles of RNA methylation in host adaptation of *P. xylostella*.

In this study, we identified *METTL3* and *METTL14* homologs in *P. xylostella* and constructed corresponding homozygous mutant strains based on CRISPR/Cas9 technology. The functional roles of *PxMETTL3* or *PxMETTL14* in the development and host adaptation were investigated based on comparing the biological parameters of each wildtype (WT) or mutant strain reared on an artificial diet (AD) and host plant. Our study provides evidence for the m^6^A-mediated trade-off between the stress adaptation to host plant defense and nutritional changes and reproduction in *P. xylostella*, which facilitates a better understanding of the mechanisms underlying the adaptative responses of insect herbivores to host plants.

## 2. Results

### 2.1. m^6^A Modification in P. xylostella

**Figure 1:**
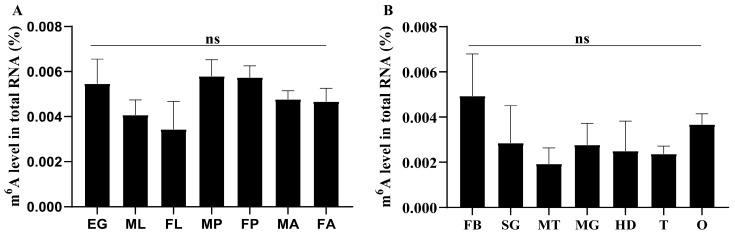
The m^6^A levels of *P. xylostella*. The m^6^A levels in different developmental stages (**A**) and tissues (**B**) are presented. EG, Egg; ML, male larva; FL, female larva; MP, male pupa; FP, female pupa; MA, male adult; FA, female adult; HD, head; MT, Malpighian tubules; MG, midgut; FB, fat body; SG, silk gland; T, testis; O, ovary. The “ns” indicates no significant difference (*p* > 0.05).

The absolute level of m^6^A in total RNA from different developmental stages and different tissues of *P. xylostella* was examined. Using the colorimetric m^6^A quantification strategy, it was found that m^6^A modifications existed in all the developmental stages and tissues tested (Figure 1A,B); however, the content was very low, and there were no significant differences.

### 2.2. Molecular Characteristics of PxMETTL3 and PxMETTL14

**Figure 2:**
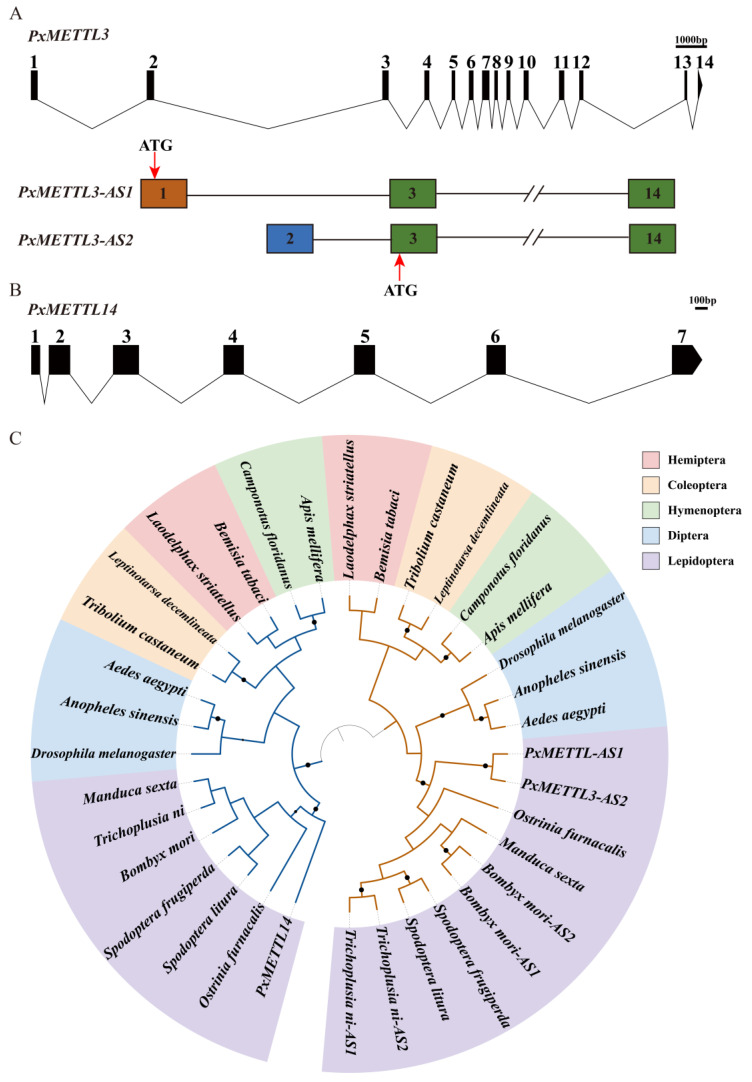
Characterization of *PxMETTL3* and *PxMETTL14*. (**A**) Genomic structure and alternative splicing of *PxMETTL3*. (**B**) Genomic structure of *PxMETTL14*. (**C**) Phylogenetic tree of insect METTL3s and METTL14s. The translation start site (ATG) is marked with an asterisk. The orange branch indicates *METTL3*s, and the blue branch indicates *METTL14*s. The circles in the tree represent bootstrap values > 0.8.

Based on the *P. xylostella* genome data and PCR results, *PxMETTL3* and *PxMETTL14* were identified as having seven and 14 exons, respectively (Figure 2A,B). Furthermore, two *METTL3* and one *METTL14* transcripts were identified, which were designated *PxMETTL3-AS1*, *PxMETTL3-AS2*, and *PxMETTL14*, respectively. The difference between the two *PxMETTL3* transcripts was located at the 5′ region, where translation was predicted to start at exon 1 of *PxMETTL3-AS1* and at exon 3 of *PxMETTL3-AS2*. The coding sequences (CDSs) of *PxMETTL3-AS1*, *PxMETTL3-AS2*, and *PxMETTL14* were 1728 bp, 1464 bp, and 1134 bp in length, respectively. At the N-terminus, 88 amino acids (aa) were lost in PxMETTL3-AS2. Domain prediction using the CDD website of NCBI revealed that PxMETTL3 and PxMETTL14 belong to the MT-A70 family. The predicted aa sequences encoded by *PxMETTL3* and *PxMETTL14* were used to construct a phylogenetic tree with 15 other insect species (Figure 2C). Among different species, *METTL3s* and *METTL14s* diverged into two branches. In Lepidoptera, *PxMETTL3* and *PxMETTL14* of *P. xylostella* are primitive in terms of their evolutionary relationship.

**Figure 3:**
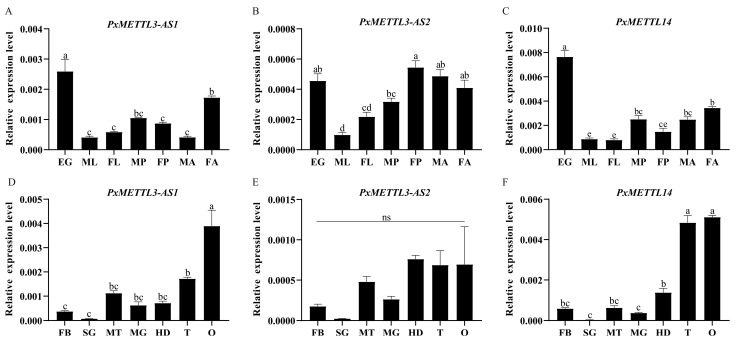
The relative expression levels of *PxMETTL3* and *PxMETTL14*. The expression levels of *PxMETTL3-AS1*, *PxMETTL3-AS2*, and *PxMETTL14* in different developmental stages (**A**–**C**) and tissues (**D**–**F**) are presented. EG, Egg; ML, male larva; FL, female larva; MP, male pupa; FP, female pupa; MA, male adult; FA, female adult; HD, head; MT, Malpighian tubules; MG, midgut; FB, fat body; SG, silk gland; T, testis; O, ovary. The “ns” indicates no significant difference (*p* > 0.05) and different lowercase letters indicate a significant difference (*p* < 0.05).

Primers were designed in specific regions of the corresponding transcripts, and qRT–PCR assays were performed. The results show that *PxMETTL3-AS1* was expressed at higher levels than *PxMETTL3-AS2*. *PxMETTL3-AS1* showed a pattern of high expression in egg and adult stages and low expression in larva. The expression of *PxMETTL3-AS1* in female adults was higher than in male adults (Figure 3A). In different tissues, the expression level of *PxMETTL3-AS1* in the ovary was higher than that in the testis, and the expression levels in the larval head, Malpighian tube, and the midgut were higher than those in the fat body and silk gland (Figure 3D). The expression levels of *PxMETTL3-AS2* were relatively high in the egg, pupa, and adult stages, with the lowest levels in the larvae, and the expression level in female pupae was higher than that in male pupae (Figure 3B). There were no differences in the expression level of *PxMETTL3-AS2* among different tissues (Figure 3E). The expression of *PxMETTL14* was the highest in eggs (Figure 3C). There was no significant difference in the expression levels of *PxMETTL14* in the adult testes and ovaries, and the expression levels in the larval fat body, Malpighian tube, and the head were higher than those in the silk gland and midgut (Figure 3F).

### 2.3. Mutant Strains of PxMETTL3 and PxMETTL14

**Figure 4:**
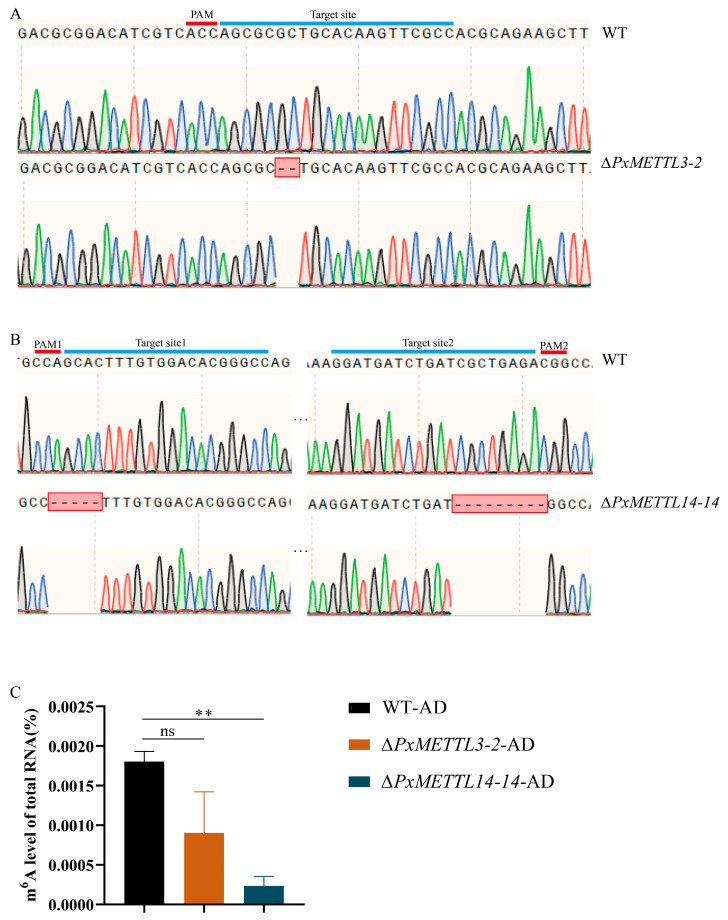
Detection of *PxMETTL3* and *PxMETTL14* mutants and changes in m^6^A levels. Schematic diagrams of two sgRNA target sites are presented (**A**,**B**). The sgRNA-targeting and PAM sequences are highlighted in red and blue, respectively. *PxMETTL3* and *PxMETTL14* mutant sequences were confirmed by cloning and sequencing. (**C**) m^6^A level of total RNA. WT-AD, wildtype individuals of the artificial diet strain; Δ*PxMETTL3-2*, Δ*PxMETTL3-2* mutant strain generated from WT-AD; Δ*PxMETTL14-14*, Δ*PxMETTL14-14* mutant strain generated from WT-AD. The “ns” indicates no significant difference (*p* > 0.05) and ** means significant level at *p* < 0.01.

A total of 222 and 256 freshly laid eggs of the AD strain were injected with a mixture of one sgRNA-METTL3 (Figure 4A) or two sgRNA-METTL14s (Figure 4B) with Cas9 protein to introduce mutations in *METTL3* or *METTL14*, respectively. The corresponding egg hatching rates were 8.11% (18/222) and 11.6% (30/256), and all larvae successfully developed into adults (G_0_). Subsequently, 5.6% (1/18) and 10% (3/30) of the adults had *METTL3* and *METTL14* mutations, respectively. The mutant adults of the same gene were mated using a single-pair strategy to establish homozygous single-mutant strains of Δ*PxMETTL3-2* (with a 2-bp deletion) or Δ*PxMETTL14-14* (with a 14-bp deletion) (Figure 4A,B). After screening for seven generations and a total of more than 1600 individuals, only 14 double-mutant homozygotes were obtained from the offspring produced by crossing the homozygous mutant strains of *PxMETTL3* and *PxMETTL14*, and only three pairs of these were successfully mated, although no offspring were produced.

To verify the functions of the *PxMETTL3* and *PxMETTL14* genes, total RNA from WT and mutant female adults was collected, and the relative m^6^A level of total RNA was measured. Compared with the WT, the relative content of m^6^A in the mutants decreased, with a significant difference in Δ*PxMETTL14-14* (Figure 4C).

### 2.4. Comparison of the Performance of WT and Mutant Strains Undergoing Host Transfer

**Figure 5:**
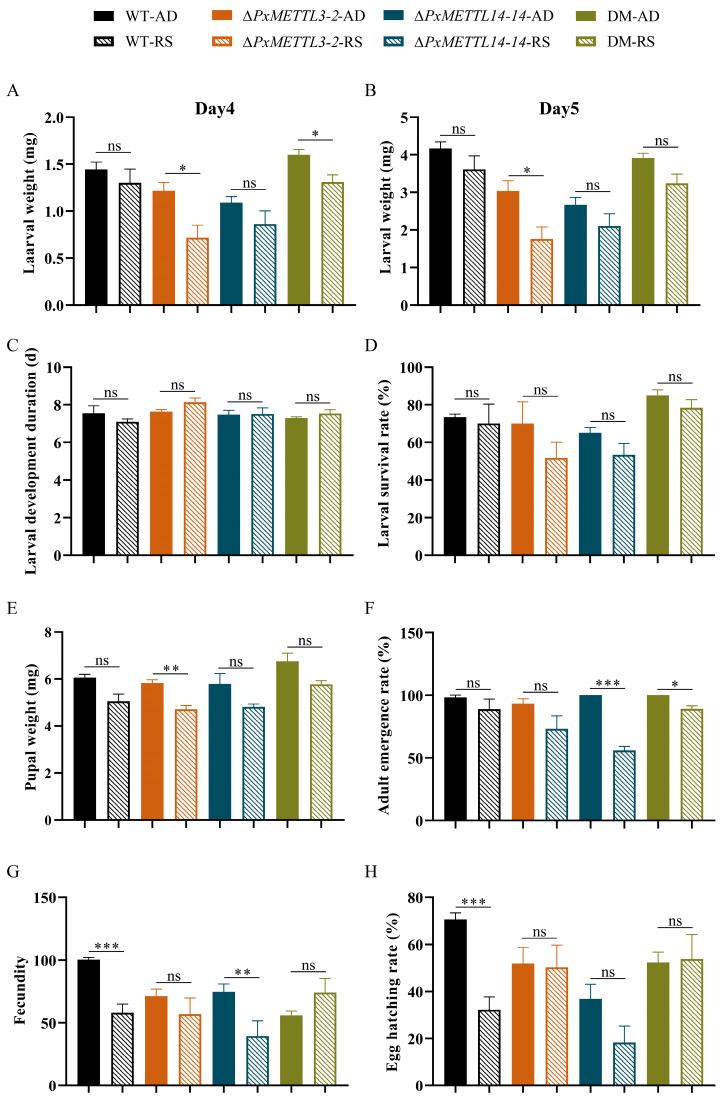
Performance of the WT and different mutant strains undergoing host transfer. The performance parameters were recorded for the larval weight on Day 4 (**A**) and Day 5 (**B**), and for larval development duration (**C**), larval survival rate (**D**), pupal weight (**E**), adult emergency rate (**F**), fecundity (**G**), and egg hatching rate (**H**). WT, Wildtype; *ΔPxMETTL3-2*, mutation of *PxMETTL3* with 2-bp deletion; *ΔPxMETTL14-14*, mutation of *PxMETTL14* with 14-bp deletion; DM, hybrid of *PxMETTL3* and *PxMETTL14*; AD, feeding on artificial diet; RS: feeding on radish seedling. The “ns” above the column indicates no significant difference between the two treatment groups (*p* > 0.05), * means significant level at *p* < 0.05, ** means significant level at *p* < 0.01, and *** means significant level at *p* < 0.001.

To study the role of m^6^A in the adaptative process of *P. xylostella* on host plants, we transferred the newly hatched larvae of the WT, each of the two mutant strains, and their hybrid offspring to feed on radish seedlings, and compared the corresponding biological parameters of the whole life cycle with those reared on an artificial diet. The results show that under the stress of host plant defense, the larval weight on Days 4 and 5 after transfer (Figure 5A,B), larval development period (Figure 5C), larval survival rate (Figure 5D), pupal weight (Figure 5E), and adult emergence rate (Figure 5F) of the WT strain were not significantly different compared to those fed the artificial diet. On the other hand, we found that the mutant strains of *P. xylostella* exhibited different degrees of adaptability when subjected to host plant defense mechanisms. Specifically, Δ*PxMETTL3-2* showed a significant reduction in larval weight at both time points tested (Figure 5A,B), and pupal weight (Figure 5E) after transferring to radish seedlings, and there were no significant differences in larval development duration, larval survival rate, or adult emergence rate (Figure 5C,D,F). Upon transfer to radish seedlings, Δ*PxMETTL14-14* showed no significant differences in larval weight, larval developmental duration, total larval survival, or pupal weight (Figure 5A–E), while the adult emergence rate of Δ*PxMETTL14-14* was significantly lower (Figure 5F). Hybrids of the *PxMETTL3* and *PxMETTL14* strains displayed significantly lower larval weights on Day 4 after transfer, as well as adult emergency rates, but had no significant effect on other aspects.

Notably, in terms of reproduction, the fecundity and egg hatching rate of the WT strain were significantly reduced when they were transferred from the artificial diet to the environment containing defense of the host plant (Figure 5G,H). There was no significant difference in fecundity and egg hatching rate of the mutant strains upon transfer from the artificial diet to the host plant, except for the significantly decreased fecundity in Δ*PxMETTL14-14*.

### 2.5. Changes in PxMETTL3 and PxMETTL14 and m^6^A Upon Host Transfer

**Figure 6:**
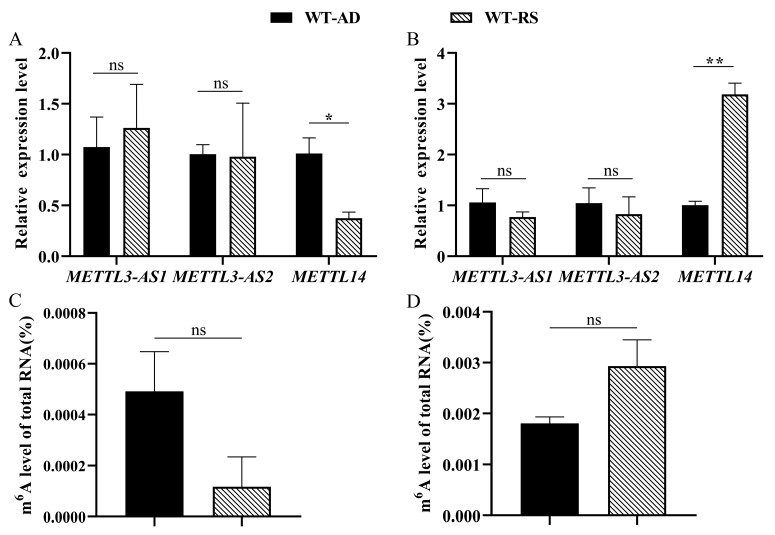
Expression of *PxMETTL3* and *PxMETTL14* and the changes in m6A levels upon host transfer. The expression levels of *PxMETTL3* and *PxMETTL14* in the larval (**A**) and female adult (**B**) stages, and the m^6^A levels in the larval (**C**) and female adult (**D**) stages are presented. The “ns” means no significant difference between different treatment groups (*p* > 0.05), * means significant at *p* < 0.05, and ** means significant at *p* < 0.01. WT, Wildtype; AD, feeding on artificial diet; RS, feeding on radish seedlings.

Based on the bioassays for the WT, or mutant strains reared on an artificial diet and transferred to the host plant, we selected the 4th-instar larvae and mature female adults for further study. The qRT–PCR results show that at the larval stage, the expression level of the *PxMETTL3* gene did not significantly change after transfer to radish seedlings, while the expression of the *PxMETTL14* gene decreased significantly (Figure 6A). At the female adult stage, the expression of *PxMETTL3* showed no significant difference after host transfer, while the expression of *PxMETTL14* increased significantly (Figure 6B). Similarly to the expression of *PxMETTL14*, the relative level of m^6^A also showed a downward trend at the larval stage (Figure 6C), and on the contrary, its level showed an upward trend at the female adult stage (Figure 6D). This may indicate that when the host environment of *P. xylostella* changes, *PxMETTL14* could respond effectively, possibly associated with the change in m^6^A levels.

### 2.6. Transcriptome and Epitranscriptome Dynamics during Host Transfer

**Figure 7:**
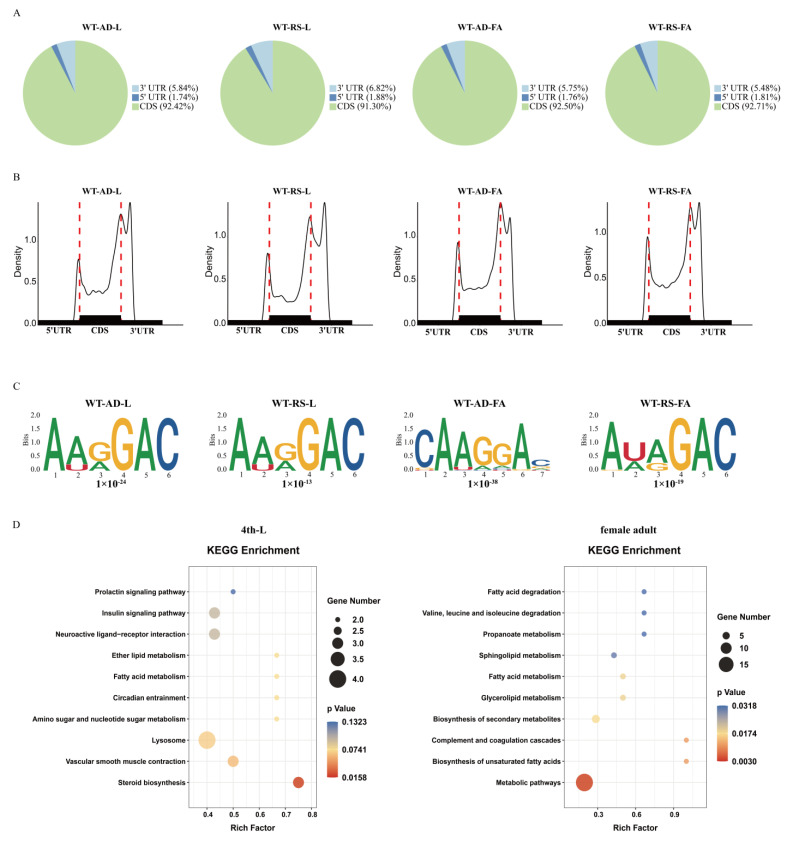
m^6^A feature and KEGG enrichment of the genes associated with host transfer in *P. xylostella*. (**A**,**B**) Distribution of the m^6^A peak in different regions of the genes. (**C**) Motifs of the m^6^A binding site. (**D**) Enrichment analysis of transcripts undergoing differential expression and epigenetic modification in the 4th-instar larvae and female adults upon host transfer. WT-AD, Wildtype individuals reared on the artificial diet strain; WT-RS, WT-AD individuals reared on radish seedlings, L: The 4th-instar larva; FA, female adult.

A series of m^6^A-immunoprecipitation (IP) and matched input (non-IP control) libraries were constructed and sequenced in order to obtain the transcriptome-wide m^6^A map for *P. xylostella*. This series included the 4th-instar larvae and mature female adults feeding on an artificial diet and transferred to radish seedlings. The transcript was divided into three segments, 5′ UTR, CDS, and 3′ UTR, and the distribution of m^6^A peaks in each segment was counted. We found that the m^6^A peaks in *P. xylostella* were abundant in the CDS (Figure 7A), mainly enriched in the start codon and stop codon regions (Figure 7B). It was estimated that in *P. xylostella*, each expressed transcript has 0.28–0.43 m^6^A peaks. We observed that the m^6^A peaks contained the canonical motif AA/UG/AGAC or CAAGGAC (Figure 7C). KEGG pathway enrichment analysis for the differentially expressed transcripts with changes in m^6^A level (with a cutoff of 1.5-fold change) revealed that the potential functions of these genes were involved in steroid biosynthesis for larval performance and metabolic pathways for adult reproduction such as fatty acids, lipids, amino acids and secondary metabolites (Figure 7D).

## 3. Discussion

The epigenetic modification of m^6^A on mRNA is of biological importance, although little is known regarding the m^6^A-mediating host plant adaptation of insect pests. In this study, we found a low level of m^6^A modification in *P. xylostella*, a specialist among cruciferous plants, and identified two RNA methyltransferase genes, *PxMETTL3* and *PxMETTL14*. A gene knockout experiment revealed that m^6^A modification may be responsible for the tradeoff between stress adaptation and reproduction of *P. xylostella* during host transfer from AD to the host plant. The genes subjected to such regulation were mainly related to steroid biosynthesis in the 4th-instar larvae and metabolic pathways in the female adults.

m^6^A is ubiquitous in eukaryotes [33], and this kind of modification regulates important cytological processes [13,14]. In different species, m^6^A occurs at relatively low levels. For example, the m^6^A modification rate was reported to be 1 to 15 m^6^A sites per RNA molecule in viruses [34], 0.7–0.9% in the yeast species *Saccharomyces cerevisiae* Meyen [3], 0.05–0.07% in *A. thaliana* [4,35], and 0.1–0.4% in mammals [36]. Based on the colorimetric m^6^A quantification strategy, we found a very low level of m^6^A modification in *P. xylostella,* accounting for only 0.002–0.008%, compared with 0.01–0.02% for *B. mori* [26]. However, based on high-throughput sequencing, we estimated that in *P. xylostella*, each expressed transcript has 0.28–0.43 m^6^A peaks, which is comparable with those in *Anopheles sinensis* Wiedemann (0.7–0.8) [37]. This indicates that the results of different methods for detecting m^6^A content may vary to different degrees, and that mRNAs of *P. xylostella* may possess universal and functional m^6^A modifications.

Two RNA methyltransferase genes *PxMETTL3* and *PxMETTL14*, were identified in *P. xylostella.* As identified in other insect species, *PxMETTL3* has two transcripts, with the expression level of *PxMETTL3-AS1* higher than that of *PxMETTL3-AS2*. The only difference between the two predicted protein sequences encoded by these two transcripts was the loss of 88 aa in the N-terminus of the PxMETTL3-AS2 protein. These genes all showed the characteristics of high expression in the egg, pupa, and adult stages, which is similar to the results of previous studies on *fl(2)d* [28]. Meanwhile, *PxMETTL3-AS1* also showed higher expression in female than in male adults, similar to the expression mode of methyltransferase VIRMA in *A. sinensis* [37]. Furthermore, we used CRISPR/Cas9 technology to knock out *PxMETTL3* or *PxMETTL14*. Interestingly, the m^6^A level of Δ*PxMETTL3-2* female adults did not decrease significantly compared with the WT, while the m^6^A level in Δ*PxMETTL14-14* decreased significantly. Upon host transfer, the expression level of *PxMETTL14* was significantly changed and closely associated with the change in m^6^A content. Coincidentally, both in human HeLa and 293FT cells, knockdown of METTL14 had a greater effect on the decrease in m^6^A levels than METTL3 knockdown [38]. The average level of m^6^A in an insect vector of rice virus, *L. striatellus*, decreased when *METTL3* and/or *METTL14* were knocked down [23]. Since it has been verified that METTL3 is a catalytically active subunit, and that METTL14 acts as an RNA-binding scaffold in the METTL3–METTL14 complex [39,40], the role of METTL14 in m^6^A modification should be reconsidered. In addition, possibly due to the existence of various methyltransferases [16], which may compensate for the function of METTL3 or METTL14, there is also a certain amount of m^6^A in mutants.

Moreover, mutations of *METTL3* or *METTL14* in insects can cause obvious phenotypic changes, and the knockdown of either of the two genes can influence important biological traits that are under the control of m^6^A modification. In *D. melanogaster*, deletion of the methyltransferase *METTL3* homolog *Ime4* resulted in a shortened lifespan, multiple behavioral deficiencies, and severe impairment in flight and locomotion. *METTL14*-deficient flies have normal wings but are also defective in flight. The double-mutant strain exhibited a phenotype similar to that of the *Ime4* mutant but with increased severity of behavioral deficiency [21]. Furthermore, both *Ime4* and *Mettl14* mutant strains show a decrease in male-specific transcripts and an increase in female-specific transcripts of *Sxl*, a master regulator of sex determination in *D. melanogaster* [20,21,22]. There is a high-frequency point mutation (A-206T) in the 5′ UTR of *CYP4C64* in thiamethoxam-resistant *B. tabaci* that brings a m^6^A site to form a m^6^A modification that increases *CYP4C64* expression, thereby conferring insecticide resistance. Knockdown of *METTL3* or *METTL14* resulted in decreased *CYP4C64* expression and increased susceptibility to thiamethoxam [24]. In BmN cells of *B. mori* infected with nucleopolyhedrovirus, the expression of the viral structural protein VP39 was increased in response to knockdown of *BmMETTL3* and *BmMETTL14*, while overexpression of *BmMETTL3* and *BmMETTL14* decreased the expression of VP39, indicating that m^6^A modification might be an epigenetic mechanism in regulating viral infection [27].

In the current study, based on a host transfer system for *P. xylostella*, we show that offspring of the WT strain reared on AD without host plant challenge could quickly adapt to the new host environment containing plant defense and nutritional stress, but the reproductive capacity of corresponding adults declined. When the mutant strains of *PxMETTL3* and *PxMETTL14* were used for the host transfer study, we found that, under the challenge of the host plant, growth and development of the larvae was significantly impaired, while the decline in adult fecundity was much lower than that of WT. Taking into account the difference between AD and radish cotyledons on defensive responses and nutritional levels, there seems to be a tradeoff between stress adaptation and reproduction in insects, which has been observed in many other biological interactions. For example, activation of the immune response reduces insect reproductive capacity [41]. With limited resources, plants respond to a variety of environmental challenges by regulating the allocation of resources for growth and defense [42], and plants often confront a decrease in yield when immunity is activated [43]. A UDP-glycosyltransferase (UGT76B1) in *A. thaliana* modulates the ratio of N-hydroxy-pipecolic acid (NHP) to NHP glycoside (NHPG), thus balancing plant growth and defense [44]. The *Wsm1* gene confers resistance to the wheat streak mosaic virus (WSMV), but leads to a reduction in yield of wheat (*Triticum aestivum* L.) [45]. Through gene expression profiling and integrative analysis of transcriptomic and epitranscriptomic data, we further found that *PxMETTL14* could respond to host plant defense effectively, and was associated with changes in m^6^A content, and steroid biosynthesis and metabolic pathways might be involved in regulating larval performance and adult reproduction, respectively. Impaired steroid production and release in *D. melanogaster* prothoracic glands leads to developmental delay and precocity [46]. In mammals, the potential role of m6A modification in the regulation of reproductive hormone secretion was found to be linked with steroid biosynthetic processes [47]. On the other hand, the mobilization of energy is tightly coupled to a variety of metabolic pathways, in which the fat body coordinates insect growth with metamorphosis or reproduction by storing or releasing key elements associated with these events [48].

**Figure 8:**
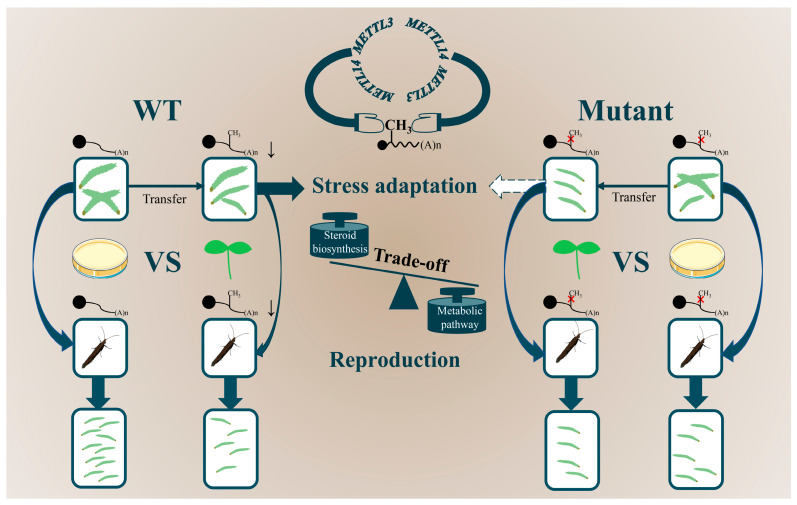
A schematic representation of m^6^A-mediated adaptation of *P. xylostella* to host plants. After the WT strain was transferred from AD to feed on radish seedlings, m^6^A modification occurred on certain sites of mRNA transcripts. This may result in expression regulation of the genes related to adaptation to host plant defense and nutritional stress through the steroid biosynthesis pathway; however, this may also result in energy reallocation through the metabolic pathway and at the expense of adult reproduction. Therefore, although the WT larval performance on the host plant was not significantly affected, WT adult reproduction was significantly reduced. Due to the lack of methyltransferase (s), the mutant strains could not regulate gene expression through m^6^A modification. Therefore, the mutant larval performance on the host plant was significantly impaired, while the mutant adult reproduction was not as affected as WT. Larvae of different sizes are proportional to their weights. Downward arrows indicate the downregulation of gene expression, and dotted arrows indicate reduced stress adaptability. The solid circle indicates the 5′ cap structure of mRNA, and “(A)n” indicates the 3′ polyA tail.

Based on the above results, we propose a model to show that m^6^A is involved in balancing the stress adaptation and the reproduction of *P. xylostella* (Figure 8). Our findings reveal an epigenetic regulation mechanism for the fast adaptation of *P. xylostella* to variable host environments and provided a comprehensive view of the multifunctional roles of m^6^A in insect-plant interactions, which paved ways for the identification of new targets for pest management.

## 4. Materials and Methods

### 4.1. Insect Strains and Host Plant

The AD strain was reared at the Institute of Applied Ecology of Fujian Agriculture and Forestry University since 2017 for more than 100 generations [49], which is the WT resource for experiments on gene knockout and host transfer. Insects were reared at 25 ± 1 °C, 65 ± 5% RH, and 16:8 h (light:dark), and larvae were fed with an artificial diet containing 20 g yeast powder, 6 g agar, 37.5 g raw wheat germ, 1 g vitamin premix, 1 g potassium sorbate, 1 g methyl paraben, 1 g ascorbic acid, 10 g sucrose, 3 g powdered radish seeds (Nanpan Prefecture), 1 mL canola oil, and 0.1 mL linoleic acid in 250 mL water [49]. Adults were fed with a 10% honey solution. The individuals of *P. xylostella* had been reared on AD without powdered radish seeds for three successive generations before they were used for the assay of host transfer.

The cruciferous host plant radish (*R. sativus*) was used. Seeds were selected from the Nanpan Prefecture white radish line and planted in rectangular plastic trays (420 mm × 320 mm × 100 mm). Plants were kept in an artificial climate box at 23 ± 1 °C and 65 ± 5% RH under a 16:8 h (light:dark) photoperiod. Radish seedlings were used for feeding insects when the cotyledon was fully stretched (approximately 1-week-old). The cotyledons of radish have been proven by our laboratory to be a suitable plant tissue for feeding *P. xylostella* since 2004 [50,51], and the growth and development of the individuals look normal and healthy.

### 4.2. Extraction of RNA and Gene Cloning

Total RNA was isolated from five female adults using a FastPure^®^ Cell/Tissue Total RNA Isolation Kit V2 (Vazyme, Nanjing, China). The extracted RNA was converted to cDNA using FastKing gDNA Dispelling RT SuperMix (Tiangen, Beijing, China). Based on the sequences of *METTL3* and *METTL14* in the *P. xylostella* genome database [50], the CDSs of *PxMETTL3* and *PxMETTL14* were cloned by PCR from female adult cDNA of the AD strain. The full-length cDNA sequences of *PxMETTL3* and *PxMETTL14* were obtained by rapid amplification of cDNA ends (RACE) using the SMARTer^®^ RACE 5′/3′ Kit (Takara, Kusatsu, Japan). PCR was performed under the following conditions: initial denaturation at 95 °C for 3 min, 35 cycles of 95 °C for 30 s, 50–60 °C for 30 s, 72 °C for 1–2 min, and a final elongation step at 72 °C for 5 min. The amplified products were confirmed by Sanger sequencing after subcloning into the pJET1.2 vector (Thermo Fisher Scientific, San Jose, CA, USA). The primers are listed in Table S1. Structural domain analysis was performed using the NCBI conservative domain database (CDD).

### 4.3. Construction of Phylogenetic Tree

The amino acid sequences of METTL3 and METTL14 of other insect species were downloaded from NCBI based on BLAST using PxMETTL3 and PxMETTL14 as queries. The amino acid sequences of insect METTL3s and METTL14s were aligned using ClustalW implemented in the MEGA 11 program. Molecular phylogenetic analyses were conducted using the neighbor-joining method, and the reliability of the tree was tested by bootstrap analysis with 1000 replications.

### 4.4. qRT–PCR

Total RNA was extracted from five individuals of different developmental stages or 100 mg eggs/tissues. Primers were designed to amplify a 90- to 200-bp fragment Table S1. The total reaction volume of 20 μL consisted of 2 μL diluted cDNA, 10 μL PerfectStart Green qPCR SuperMix (Transgene, Beijing, China), 0.4 μL of each primer, and 0.4 μL of CXR dye. Samples were run on an ABI Q6 real-time system (Applied Biosystems, Bedford, MA, USA) using the following temperature cycling conditions: 30 s of activation at 94 °C, followed by 40 cycles of 5 s at 94 °C, 15 s at 60 °C, and 10 s at 72 °C. The expression level was calculated using the 2^−ΔΔCt^ method based on three biological replicates. The ribosomal protein L32 (RPL32) of *P. xylostella* was used as the reference gene.

### 4.5. Design of sgRNA and Off-Target Analysis

The sgRNA targeting sites were selected according to the GGN_18_NGG [52] principle, and were set on exon 3 of *PxMETTL3* and exon 3 of *PxMETTL14*. The online website Cas-OFFinder (http://www.rgenome.net/cas-offinder/ (accessed on 15 October 2020)) was used to predict off-target effects. The primers for detecting mutations were designed based on the genome sequence Table S1.

### 4.6. Preparation of sgRNA and Embryo Microinjection

The preparation of sgRNA includes two components [53]. The PCR template and PCR program parameters were based on those recommended in the manual of Phanta Max Super-Fidelity DNA Polymerase (Vazyme, Nanjing, China). PCR products were purified by an OMEGA Gel Extraction Kit (OMEGA, Atlanta, GA, USA) and then used for in vitro transcription using the MEGAscript™ T7 High Yield Transcription Kit (Thermo Fisher Scientific, San Jose, CA, USA) according to the manufacturer’s instructions. The Cas9-N-NLS nuclease was purchased from GenScript Biotech Company (Piscataway, NJ, USA).

The mixture containing 100 ng/μL sgRNAs and 200 ng/μL Cas9 protein was incubated at 37 °C for 15 min, and then used for embryo microinjection. After injection, the egg cards were placed in a sterile petri dish with a sterile wet paper towel in the middle. After 24 h, egg cards were transferred to a box with enough feed for further incubation.

### 4.7. Establishment of Mutant Strains

The larvae that emerged after injection were called the G_0_ generation. Each adult of the G_0_ generation was paired with one WT adult of the opposite sex separately. After mating and spawning, DNA of the G_0_ generation was extracted using a TIANamp Genomic DNA Kit (Tiangen, Beijing China). PCR amplification based on specific detection primers was used to detect gene mutations at the target sites Table S1.

When a G_0_ adult was shown to be edited, its G_1_ offspring was randomly inbred through single-pair mating to produce G_2_ offspring. Strict inbreeding screening continued until a homozygous mutant appeared. Homozygous mutant strains were isolated, propagated, and randomly detected for mutations in each generation.

*PxMETTL3* and *PxMETTL14* are known to be located on different chromosomes, and an attempt was made to obtain the double-mutant strains by means of a cross-screening. However, it was found in the follow-up test that the mating of homozygous double-mutant adults could not produce offspring. Therefore, the heterozygous offspring derived from the cross of mutant strains of *PxMETTL3* and *PxMETTL14* were used in the studies.

### 4.8. Quantification of m^6^A

The m^6^A level in total RNA was measured using the EpiQuik™ m^6^A RNA Methylation Quantification Kit (EpiGentek, Farmingdale, NY, USA) as recommended by the manufacturer. To determine the m^6^A RNA methylation status of two different RNA samples, the relative percentage of m^6^A in the total RNA was calculated using the following formula: m^6^A %$=\frac{\left(\mathrm{Sample}\;\mathrm{OD}-\mathrm{NC}\;\mathrm{OD}\right)÷S}{\left(\mathrm{PC}\;\mathrm{OD}-\mathrm{NC}\;\mathrm{OD}\right)÷P}\times 100\%$. The absolute percentage of m^6^A in the total RNA can be calculated using the following formula: m^6^A %$=\frac{\mathrm{Sample}\;\mathrm{OD}-\mathrm{NC}\;\mathrm{OD}}{\mathrm{Slop}*\mathrm{Total}\;\mathrm{RNA}\;\left(\mathrm{ng}\right)}\times 100\%$, where “OD” indicates optical density, “NC” indicates negative control, “PC” indicates positive control, “S” is the amount of input sample RNA in ng, and “P” is the amount of input PC in ng. The slope was calculated using the most linear part of the standard curve.

### 4.9. Insect Bioassays

To verify whether the gene mutation of *P. xylostella* has any influence on host adaptability, 20 newly hatched larvae of the WT and mutant strain were placed on radish seedlings with fully stretched cotyledons, and three biological replicates were set for each strain.

The larval weight on Days 4 and 5 after treatment, the larval development duration, and the larval survival rate until late 4th-instar were recorded. The 4th-instar larvae were also used to evaluate the gene expression, m^6^A content, transcriptome, and epitranscriptome because this is the most destructive developmental stage when *P. xylostella* consumes a large amount of host plant. The pupal weight of newly emerged pupae as well as the number of successfully emerged adults were counted. The pupae were removed from the radish seedlings and single individual was kept in one 1.5 mL EP tube (Biosharp, Shanghai, China) for eclosion. To test the reproductive capacity, single-pair WT or mutant adults, after transferring to radish seedlings, were mated, and the eggs (fecundity) were counted for 48 h. After this, the egg cards were kept and observed for the number of larvae hatched. The egg hatching rate was calculated by dividing the total number of eggs by the total number of hatching larvae.

The same biological parameters and molecular characteristics of the WT or mutant individuals reared on AD without powdered radish seeds were measured as controls and used to compare with those of the counterparts after transferring to radish seedlings.

### 4.10. m^6^A-seq and RNA-seq

The 4th-instar larvae or mature female adults of *P. xylostella* reared on an artificial diet or transferred to radish seedlings were sampled. Two biological replicates were set, and each replicate contained 15 individuals. Total RNA was isolated and fragmented into small pieces. Then, the cleaved RNA fragments were used to construct a library. The libraries were sequenced at LC-Bio (Hangzhou, China). The screening criteria of the KEGG pathway for differential genes were set as log_2_ fc > 0.584 or log_2_ fc < −0.584.

### 4.11. Statistical Analysis

Multiple datasets were compared using one-way ANOVA followed by Tukey’s HSD test. Two groups of data were compared using independent samples t tests. All data analyses were performed using SPSS software (version 25.0, Armonk, NY, USA). Data are shown as the mean ± standard error (SE).

### 4.12. Data Availability

The raw sequence data of m^6^A-seq and RNA-seq have been deposited in the Genome Sequence Archive (GSA) in the BIG Data Center operated by the Beijing Institute of Genomics (BIG), Chinese Academy of Sciences, with accession number of CRA007211, which is publicly accessible at http://bigd.big.ac.cn/gsa (accessed on 12 June 2022).

The sequences of *PxMETTL3-AS1*, *PxMETTL3-AS2*, and *PxMETTL14* have been submitted to NCBI GenBank, with accession numbers of ON754976, ON754977, and ON754978.

## Data Availability

Publicly available datasets were analyzed in this study. This data can be found here: http://bigd.big.ac.cn/gsa. Accession number: CRA007211.

## Supplementary Materials

The following supporting information can be downloaded at: https://www.mdpi.com/article/10.3390/ijms231710013/s1.
