# Supplementary material for: Functional Characterization of Two RNA Methyltransferase Genes METTL3 and METTL14 Uncovers the Roles of m6A in Mediating Adaptation of Plutella xylostella to Host Plants
Source: Int J Mol Sci. 2022 Sep 2;23(17):10013. doi: 10.3390/ijms231710013 (PMC9456542; doi:10.3390/ijms231710013)
Supplement: Supplementary file 1 [file ijms-23-10013-s001.zip › ijms-1790861-supplementary.pdf]

Supplementary Materials

Table S1. The primers used in this study.

Primer Name	Sequence (5′-3′)	Purpose
METTL3-F	GTGTTAATGTATCCTTCAAGTTTC	ORF amplification
METTL3-R	GAAATCTATTTATTGACGTCGATAC	ORF amplification
METTL3-3′RACE-1F	CCTTCAAGAAGCGCTACCCCGACGGC	ORF amplification
METTL3-3′RACE-2F	GCCCATTATCGACGTAAATAAATAGATT	ORF amplification
METTL3-5′RACE-1R	GGCGAACTTGTGCAGCGCGCTGGTGACG	ORF amplification
METTL3-5′RACE-2R	GGGCACTGTCGGCTTCTCTGTCTTTT	ORF amplification
ME14-1F	GAAGAAACTGCTCGCGCAAA	ORF amplification
ME14-1R	ACAGGAACACGAAGGCTCTG	ORF amplification
ME14-2F	CCGCAGAACTTCATCCGAGA	ORF amplification
ME14-2R	GCGTAGAGAGAGGCGTTGAA	ORF amplification
METTL14-3′RACE-1F	GGGACGGAGAAGAATCCACCTGTTCGG	ORF amplification
METTL14-3′RACE-2F	GGCTTCCGGCGCTGCGAGGACATCTG	ORF amplification
METTL14-5′RACE-1R	CCCTTCAAGAACGTAGAAGAGTCGGTG	ORF amplification
METTL14-5′RACE-2R	GGCTCCACGAGCAGCACGTCGAAC	ORF amplification
sgRNA-METTL3	TAATACGACTCACTATAGGCGAACTTGTGCAGCGCGC GTTTTAGAGCTAGAAATAGCAAGTTAAAATAAGGCTA GTCC	sgRNA synthesis
sgRNA-METTL14-1	TAATACGACTCACTATAGGCCCGTGTCCACAAAGTGC GTTTTAGAGCTAGAAATAGCAAGTTAAAATAAGGCTA GTCC	sgRNA synthesis
sgRNA-METTL14-4	TAATACGACTCACTATAGGATGATCTGATCGCTGAGA GTTTTAGAGCTAGAAATAGCAAGTTAAAATAAGGCTA GTCC	sgRNA synthesis
METTL3-test-F	GTCCTCTATTTCCCTCCCTGTAG	Deletion test
METTL3-test-R	CAATAACATTTCCAACAGCCACC	Deletion test
METTL14-test-F	GCCCCCAAAACATCTCTGCAC	Deletion test
METTL14-test-R	CCTTCAGATCACACTTCAAGTAC	Deletion test
qpcr-ME3-AS1-1F	ACAGCCTTCGAGAGAAGTTGG	qRT-PCR
qpcr-ME3-AS1-1R	CCCCTTGTCATGTCTACTGCT	qRT-PCR
qpcr-ME3-AS2-1F	CGTAGTTTCGGTCGTGCCTC	qRT-PCR
qpcr-ME3-AS2-1R	GCTAGCAGACAGGATCAACCA	qRT-PCR
ME14-qPCR-1F	ACGTTCTTGAAGGGCACACA	qRT-PCR
ME14-qPCR-1R	GTACATAGGTGGAGTGGCCG	qRT-PCR
